# A Cross-Sectional Study of Resident Training in Robotic Surgery in India

**DOI:** 10.7759/cureus.22162

**Published:** 2022-02-13

**Authors:** Danny Darlington, Fatima Shirly Anitha, Carbin Joseph

**Affiliations:** 1 Uro-Oncology, Max Super Speciality Hospital, Delhi, IND; 2 Pediatrics, Sri Ramachandra Institute of Higher Education and Research, Chennai, IND; 3 General Surgery, Kanyakumari Government Medical College and Hospital, Nagercoil, IND

**Keywords:** minimally invasive surgery, resident training, robotic surgery, quality of training, robot-assisted surgery

## Abstract

Background

The widespread implementation of robotic surgery in the Indian subcontinent has received mixed reactions from residents and mentors alike. To date, however, no study has documented the perception of Indian surgical trainees on the effect of robotic surgery on surgical training. Therefore, we conducted a questionnaire-based study on Indian surgical residents to assess their views about robotic surgery and the effect, they believe, it might have on resident training.

Materials and methods

Questionnaires were distributed to 300 surgical residents from programs that do not have surgical robots. All other residents, faculty, medical students, and interns were excluded from the study. The responses were collected and analyzed using appropriate statistical methods.

Results

Overall, 210 surgical residents responded to the survey. A majority of them (57.72%) reported low levels of knowledge regarding robotic surgery. While 88.10% of the study participants reported that the use of robotic surgery will continue to rise in India, an equal proportion (88%) believed that procuring a robot in their program will impair their training in open and laparoscopic surgeries.

Conclusions

The introduction of robotic surgery into surgical residency programs is seen, by most residents, as a threat to training in traditional surgical methods. This calls for the effective incorporation of robotic training into residency training with equal distribution of resident training cases in programs across the country.

## Introduction

Robotic surgery is one of the most significant improvements in minimally invasive surgery in recent years. With magnified three-dimensional vision and motion scale, it offers unprecedented advantages to patients and surgeons. This novel modality of surgery is increasingly being used in the Indian subcontinent. However, the high cost associated with robotic surgical procedures can be detrimental, considering the lower socioeconomic status of the diverse Indian population [1]. There is also an unmet need for robotic surgical training, as relatively few centers perform robotic surgeries in India. There is no standardized curriculum for training in robotic surgery [2]. This might induce a vicious cycle, negatively affecting resident training in robotic and minimally invasive procedures. Currently, no study is available on the training for robotic surgery in India and hence we conducted this study to analyze this among residents from Indian hospitals.

## Materials and methods

Aims and study design

Our study aimed to analyze the perception of Indian surgical residents about robotic surgical training and its effects. It was a cross-sectional, questionnaire-based study conducted between August and December 2020 in Kanyakumari Government Medical College and Hospital, Kanyakumari, Tamil Nadu, India. The study was approved by the institutional ethical committee of Kanyakumari Government Medical College and Hospital vide letter No. IEC/SC/2020/02/0071.

Inclusion and exclusion criteria

The study included only trainee residents in general surgery from different parts of India. Residents from other disciplines were excluded from the study and so were the faculty, staff grade doctors, interns, and medical students. Residents from programs with surgical robot platforms were also excluded from the study. Participation was voluntary, and the details of the participants and their responses were kept strictly anonymous.

Data collection

A validated questionnaire was personally given to 300 resident doctors in general surgery from government hospitals and medical colleges at various meetings and masterclasses held during the study period (Table 1) [3]. A summary of the study was provided, and the questionnaire was explained after obtaining informed consent from the study participants. The trainees were then encouraged to fill in their answers themselves without any consultation with others. The anonymized responses were collected back after adequate time.

**Table 1:** The self-administered questionnaire used in the study

1.	What is your gender?
2.	What is your current year of residency?
3.	What is your current level of interest in robotic surgery? High/intermediate/low
4.	What is your level of knowledge of robotic surgery? Good/intermediate/poor
5.	Have you been involved in any robotic surgeries before? Observed/assisted/performed
6.	What do you feel will happen to the trend of robotic surgery in India? Increase/remains unchanged/decrease
7.	Do you feel robot-assisted surgery will fulfill an increasingly important role in surgical specialties? Yes/no
8.	Do you feel the use of robotic surgery is feasible within the Indian healthcare system? Yes/no
9.	Do you feel the use of robotic surgery is superior to traditional open surgical techniques? Yes/no
10.	Do you feel the use of robotic surgery is superior to laparoscopic surgical techniques? Yes/no
11.	Do you feel robotic surgery will become the new gold standard for certain surgical procedures in specialties like urology? Yes/no
12.	Do you feel you will use robot-assisted surgery during your career in the future? Yes/no
13.	Do you feel your residency program should increase its emphasis on robot-assisted surgery? Yes/no
14.	Are you interested in pursuing a fellowship in robotic surgery in the future? Yes/no
15.	Does your residency program currently have a Da Vinci surgical system? Yes/no
16.	Do you believe that increasing the number of robotic surgeries in a unit will reduce resident learning in open and laparoscopic surgeries? Yes/no
17.	Do you think hospitals with surgical robots provide superior or better health care? Yes/no
18	Do you want your residency program to procure a surgical robot? Yes/no
19.	How do you feel the procurement of a robot will affect your residency training? Beneficial/detrimental
20.	Do you like to have good exposure to robotic surgery in your surgical residency? Yes/no
21.	Which hospital setting is your current residency program based upon? State-run medical college/private medical college/corporate hospital

Statistical analysis

The questionnaire was completed by the respondents, and the data were collected and entered into Microsoft Office Excel 2010 (Microsoft Corporation, Redmond, WA). The analyses and calculations were performed using Microsoft Office Excel 2010 (Microsoft Corporation). The responses were analyzed and presented graphically using the same software. This study was performed as a cross-sectional study, and no subgroup analysis was performed because of the small sample size.

## Results

**Figure 1:**
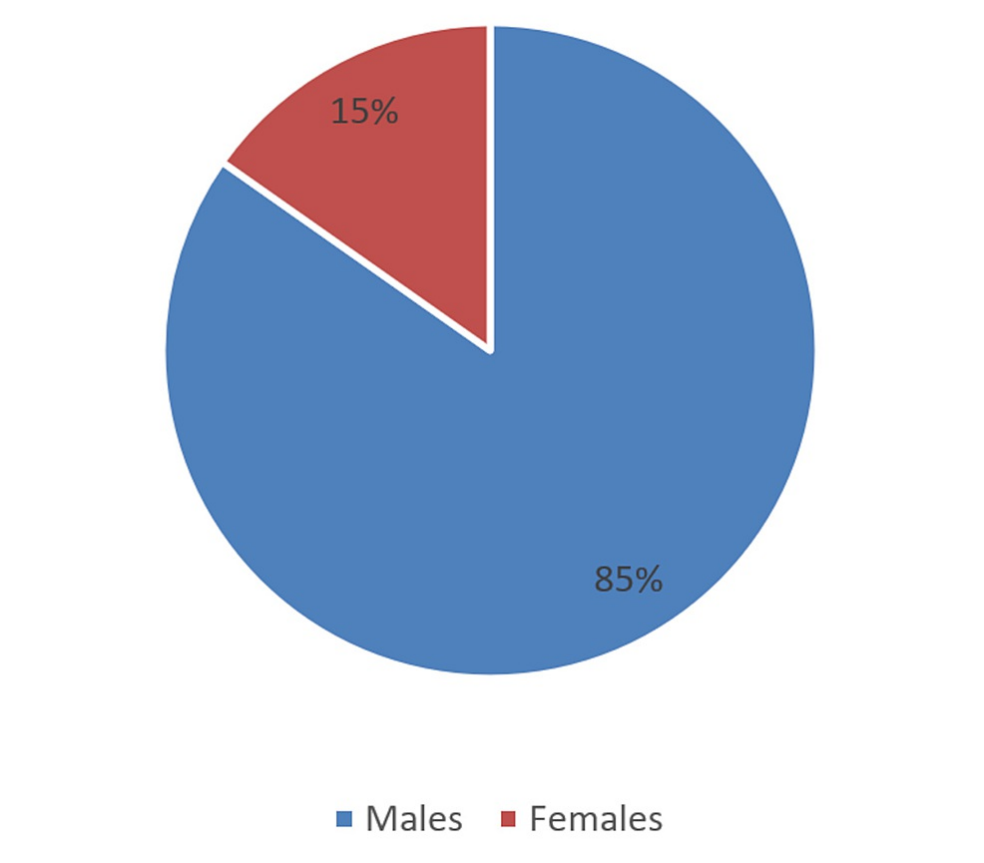
Gender distribution of the study participants

Among the 300 general surgery residents who were given the questionnaire, 210 completed the survey, a response rate of 70%. Most of the residents (200 out of 210) were in surgical residency programs in government medical colleges, comprising 95.24% of the respondents. The remaining 10 were surgical residents from corporate hospitals and formed 4.76% of the respondents. None of the residents had access to surgical robots in their residency programs. Males formed a significant proportion of the respondents (85%) in contrast to 15% female resident surgeons (Figure 1).

**Figure 2:**
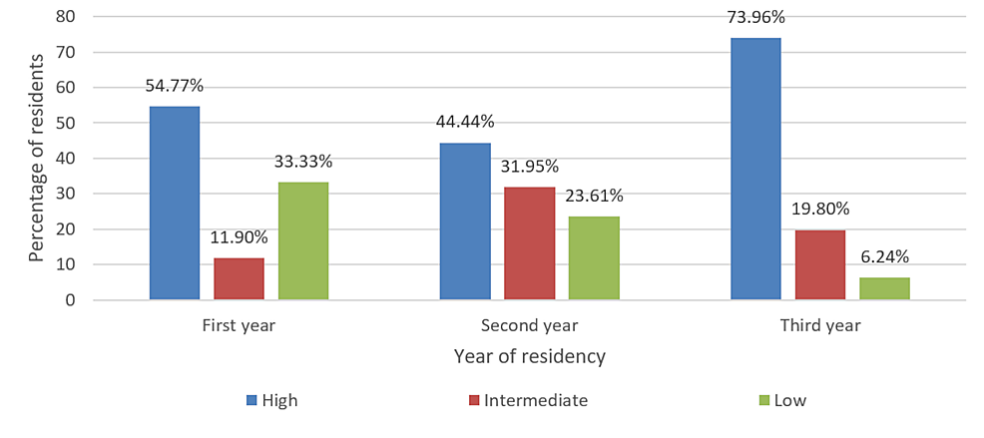
Levels of interest in robotic surgery among the study participants

**Figure 3:**
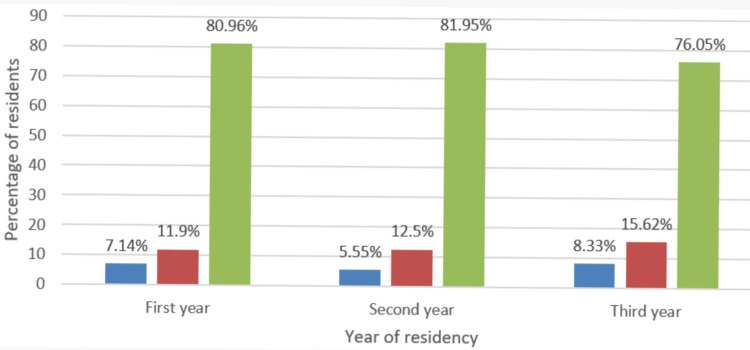
Knowledge of robotic surgery among surgery residents

**Figure 4:**
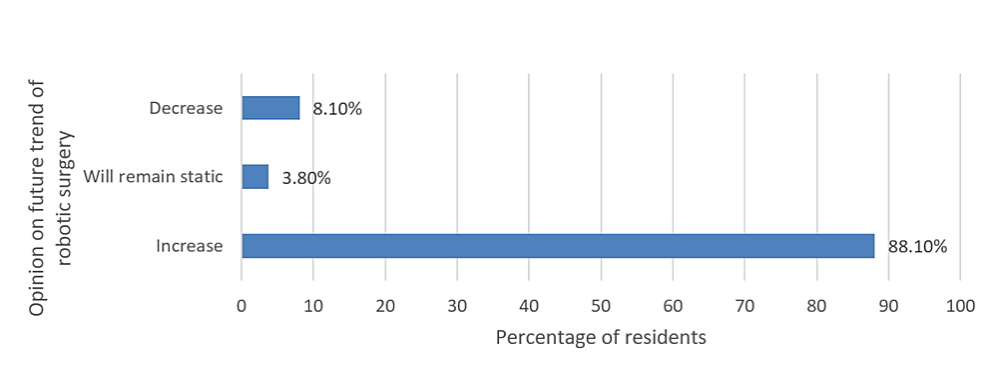
Resident opinion on the future trend of robotic surgery in India

**Figure 5:**
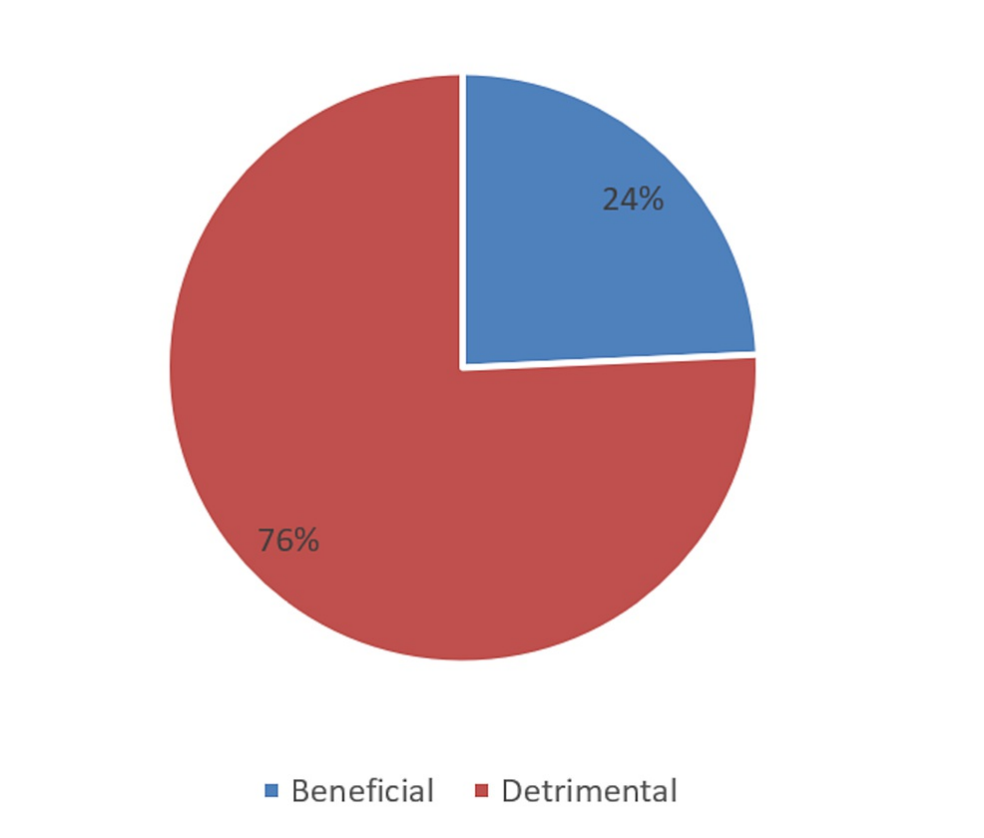
Resident perception of the effect of procuring a robot on resident training in non-robotic surgeries

Although residents universally had a high interest in learning robotic surgery, the perceived knowledge was poor in all three years of surgical residency (Figures 2-3). However, 88.10% of the respondents predicted that the use of robot-assisted surgery will continue to rise in India (Figure 4). Most of the residents felt that using robot-assisted surgery in their units will be detrimental to their training (Figure 5).

Most participants believe that robotic surgery is superior to traditional surgical methods and will be able to fulfill an important role in surgical specialties. Almost 88% of the trainees believe that procuring a robot will seriously impair resident training in other methods of surgery; however, an equal proportion of trainees were against their hospitals starting a robotic training program (Table 2).

**Table 2:** Resident responses to the questionnaire used in the study *N*=number of respondents

Questions	Yes N (%)	No N (%)
Do you feel robot-assisted surgery will fulfill an increasingly important role in surgical specialties?	192 (91.42)	18 (8.58)
Do you feel the use of robotic surgery is feasible within the Indian healthcare system?	138 (65.71)	72 (34.28)
Do you feel the use of robotic surgery is superior to traditional open surgical techniques?	167 (79.52)	43 (20.48)
Do you feel the use of robotic surgery is superior to laparoscopic surgical techniques?	174 (82.85)	36 (17.15)
Do you feel robotic surgery will become the new gold standard for certain surgical procedures in specialties like urology?	143 (68.09)	67 (31.91)
Do you feel you will use robot-assisted surgery during your career in the future?	119 (56.67)	91 (43.33)
Do you feel your residency program should increase its emphasis on robot-assisted surgery?	42 (20.00)	168 (80.00)
Are you interested in pursuing a fellowship in robotic surgery in the future?	134 (63.80)	76 (36.20)
Do you believe that increasing the number of robotic surgeries in a unit will reduce resident learning in open and laparoscopic surgeries?	185 (88.09)	25 (11.91)
Do you think hospitals with surgical robots provide superior or better health care?	148 (70.48)	62 (29.52)
Do you want your residency program to procure a surgical robot?	34 (16.20)	176 (83.80)
Do you like to have good exposure to robotic surgery in your surgical residency?	128 (60.95)	82 (39.05)

## Discussion

This study analyzes the perception of Indian surgical residents on robotic surgery. Overall, 210 residents completed the survey questions. Most residents felt that the number of robotic surgical procedures will increase in the future in India; however, they also felt insecure about resident training in traditional open and laparoscopic surgeries in centers with a surgical robot. At the same time, most residents in the study realized the significance of fellowship training in robotic surgery.

Minimally invasive surgery is increasingly being used globally due to its advantages such as less postoperative pain, early recovery, and short hospital stay. The disadvantages of laparoscopy include two-dimensional vision without depth perception, limited range of movements, difficulty in hand-eye co-ordination, and considerable experience needed to breach the learning curve. However, the introduction of robotic surgical platforms overcomes the shortcomings of laparoscopic surgery [4]. While the Da Vinci system enjoyed a monopoly in the robotic surgical market, several robotic systems have been recently introduced, and the future looks good as far as the patient and surgeon are concerned [5].

Robot-assisted surgery is carried out predominantly in private hospitals in India though few government institutions have also established robotic surgical platforms. In India, being a developing nation, the cost-effectiveness of robotic surgery has been widely debated [6,7]. Being a relatively recent field of surgery, surgeon training is important. However, there is no standardized curriculum for teaching robotic surgery in most resident training programs in India [8]. Like any other surgical technique, proficiency in robotic surgery can be achieved by breaching the learning curve, which generally requires the surgeon to have performed a minimum number of individual surgeries [9]. This underscores the impact it has on the training of future surgeons, as a large proportion of open and laparoscopic procedures performed previously by residents will be diverted to robotic surgery. In contrast, residents would benefit from getting exposed to this novel technology earlier in their careers.

Before embarking on robotic surgery, having a good and supportive team is imperative [10]. It is very important to train the surgeon as well as the operating room staff [11]. Training in robotic surgery requires access to the machine and a mentor. In most centers, the training program in robotic surgery is not standardized. There is also a definite possibility of a reduction in the number of open and laparoscopic surgeries in a robotic unit. This may, in turn, affect resident training in traditional surgeries. Hence, a curriculum for adequate training should be designed to better educate future residents in hospitals with robotic platforms. Residency training should not be limited just to observing or assisting in robotic surgeries. Hands-on console training should be encouraged right from residency training. Dual-console robots that are specifically designed for training young surgeons in robot-assisted surgery should be preferred in teaching hospitals [12]. A three-tier approach for robotic training can be followed in which the resident is given progressively complex steps in the surgical procedure [13].

Patients undergoing robotic surgery should be made to understand the advantages and disadvantages of this novel modality of surgery [14]. As the knowledge on robotic surgery increases with patient education levels, every effort should be made to educate patients, particularly those belonging to lower socioeconomic groups and lower literacy levels [15]. This calls for outreach programs and online educative sessions for the general public. Such programs should be conducted preferably by surgeons who are trained in robotic surgery.

Very few studies embark on assessing the attitude and perception of residents toward robotic surgery. In the Canadian study done by Locke et al., more than 75% of residents responded that the presence of a robot in their training program reduces residents' chances of learning open and laparoscopic procedures. Similarly, 60.9% of the residents in their study reported that the absence of a surgical robot in a teaching hospital is beneficial to residency training [3]. These findings reflect the results of our study in which 88.09% of the residents agreed that robotic surgery decreases their chances in open and laparoscopic surgeries.

To the authors' knowledge, the present study is the first conducted among Indian surgical residents on training in robotic surgery and its effects on their general training program. The study included only residents from hospitals without a robotic program. Hence, the study reflects the ideas of most government-funded surgical training centers in India, where robotic surgery is still a dream.

The study has a few limitations as well. The study did not involve residents from other surgical disciplines such as surgical gastroenterology, gynecology, and urology. Hence, the ideas may not be representative of specialty trainees in the country. Although the sample size includes residents from all over the country, the number is small and hence a comparative analysis could not be performed. Therefore, studies with a larger sample size are needed to further assess the resident perception of robotic surgery and its effects on training. We could not assess the background knowledge of the respondents about robotic surgery and therefore could only assess the residents’ perception rather than the actual effect robotic surgery has on resident training. This could be done in future studies with a larger sample size, including residents from centers with robotic platforms.

## Conclusions

Robotic surgery is being adopted in India at a modest pace. Although it offers several advantages over traditional surgical methods, its high cost is an important factor, especially in developing countries like India. A wide gap in the knowledge about robotic surgery exists in India among doctors and patients. Hence, for robotic surgery to flourish in the country, urgent interventions aimed at educating the masses are the need of the hour. As there is no standardized curriculum to train robotic surgeons, a structured residency and fellowship training should be implemented. Robotic surgery training should be integrated into the residency training curriculum so that the trainee surgeons acquaint themselves with the basics of robotic surgery in the early phase of their career. The perception of residents who are future robotic surgeons also needs to be changed. Achieving lifelong learning and career development should be the long-term goal for surgical residents in this ever-changing era of medicine.
